# Study on TPD Phasemeter to Suppress Low-Frequency Amplitude Fluctuation and Improve Fast-Acquiring Range for GW Detection

**DOI:** 10.3390/s24113434

**Published:** 2024-05-26

**Authors:** Min Ming, Jingyi Zhang, Huizong Duan, Zhu Li, Xiangqing Huang, Liangcheng Tu, Hsien-Chi Yeh

**Affiliations:** TianQin Research Center for Gravitational Physics, School of Physics and Astronomy, Sun Yat-sen University (Zhuhai Campus), Zhuhai 519082, China; mingm3@mail.sysu.edu.cn (M.M.);

**Keywords:** laser interferometer, amplitude noise suppression, tangent phase detector, phasemeter, cycle slip

## Abstract

A phasemeter as a readout system for the inter-satellite laser interferometer in a space-borne gravitational wave detector requires not only high accuracy but also insensitivity to amplitude fluctuations and a large fast-acquiring range. The traditional sinusoidal characteristic phase detector (SPD) phasemeter has the advantages of a simple structure and easy realization. However, the output of an SPD is coupled to the amplitude of the input signal and has only a limited phase-detection range due to the boundedness of the sinusoidal function. This leads to the performance deterioration of amplitude noise suppression, fast-acquiring range, and loop stability. To overcome the above shortcomings, we propose a phasemeter based on a tangent phase detector (TPD). The characteristics of the SPD and TPD phasemeters are theoretically analyzed, and a fixed-point simulation is further carried out for verification. The simulation results show that the TPD phasemeter tracks the phase information well and, at the same time, suppresses the amplitude fluctuation to the noise floor of 1 μrad/Hz^1/2^, which meets the requirements of GW detection. In addition, the maximum lockable step frequency of the TPD phasemeter is almost three times larger than the SPD phasemeter, indicating a greater fast-acquiring range.

## 1. Introduction

With the announcement of LIGO’s successful detection of gravitational waves (GWs) on the ground [1], space gravitational wave detection has become a hot topic around the world. Space-based GW detection is complementary to ground-based detection within the frequency band of 0.1 mHz~1 Hz. The international space gravitational wave detection programs mainly include LISA [2], DECIGO [3], the Taiji project [4], and the TianQin project [5]. The TianQin project, proposed by Chinese researchers, aims to launch a space-based gravitational wave observatory around 2035 [6,7,8]. Three satellites will be launched to form the space-based GW observatory in Earth’s orbit. The arm length of the interferometer will be 170,000 km. The displacement between the two satellites is read out by the phase of the heterodyne laser interferometer tone. For GW detection, the displacement accuracy should be at least 1 pm/Hz^1/2^, taking the laser frequency, RIN, TTL, etc., into consideration, and the noise of the phase readout system should be less than 0.2 pm/Hz^1/2^, which corresponds to about 1 μrad/Hz^1/2^. The phasemeter is also used in beam pointing, data communication, GNSS, and absolute distance measurement [9,10,11].

Due to the extremely high accuracy requirements of phase measurements for space GW detection, scientists have dedicated significant effort. In 2006, Pollack et al. proposed a zero-crossing method using a technique of counting and timing to measure the phase [12,13]. To improve sensitivity, Hsu et al. developed an all-digital phasemeter based on a phase-locked loop (PLL) [14]. The PLL phasemeter uses a sinusoidal characteristic phase detector (SPD) to detect the difference between the local numerically controlled oscillator (NCO) and the measurement signal. Then, the phase of the NCO is locked to the input signal by closed-loop control, which is thus a measure of the heterodyne tone. By this design, the phasemeter reaches a sensitivity of 3 μrad/Hz^1/2^ at 1 Hz and above. Such high resolution makes the SPD phasemeter a commonly used scheme for inter-satellite laser interferometers [8]. To achieve the phase measurement requirements of multi-channel and high-speed in GW detection, the advantages of a field programmable gate array (FPGA) for high-speed and parallel processing are fully utilized. Gerberding et al. built a precise model of an all-digital SPD phasemeter, hosted in an FPGA, and processed digital data in a fixed-point mode. The phasemeter achieved a performance better than 1 μcycle/Hz^1/2^@1 mHz [15]. Heinzel et al. proposed a multiple-channel algorithm to increase the signal-to-noise ratio [16]. To reduce the sampling time jitter noise, the special pilot tone technique was applied in a weak-light DPLL [17,18,19]. In our previous work, we reported a phasemeter based on an SPD phasemeter in an FPGA for the TianQin project. A least squares method is proposed to obtain an accurate correction coefficient rather than preset values by normal pilot tone correction. The noise floor reached 5 μrad/Hz^1/2^, corresponding to displacement noise of less than 1 pm/Hz^1/2^ [20]. However, the aging of electronic components, the creep of structures, and the changing contrast in the interferometer can lead to low-frequency amplitude fluctuation. The high-frequency amplitude fluctuations caused by relative intensity noise are not examined in this manuscript. In addition, heterodyne frequencies may change greatly in complex space-borne environments, so a larger fast acquisition range would be better for phase tracking and avoiding cycle slips.

To stabilize the amplitude of the heterodyne tone, using the other branch of the *I*/*Q* demodulation for automatic gain control (AGC) is discussed in Nils Christopher Brause’s essay [21]. However, the AGC relies on stable phase control to get amplitude information, which in turn is affected by the change in gain. This coupling makes AGC not an optimal option for us. Therefore, open-loop amplitude suppression with less coupling is preferred. Lee et al. proposed a digital tanlock loop (DTL) phasemeter, which uses the tan^−1^ function of the incoming signal to suppress amplitude fluctuation and has a linear characteristic, with a period of 2π [22]. Uhran and Lindenlaub carried out experimental studies of a modified nth-order tanlock system, and the results show that the tanlock system has larger lock ranges than an SPD phasemeter [23]. Therefore, applying a tangent phase detector in a phasemeter for GW detection will make it possible to obtain better amplitude fluctuation suppression and a larger locking range.

Overall, the phasemeter requires high accuracy, insensitivity to amplitude fluctuation, and preferably a large lock range. The traditional SPD phasemeter has the advantages of a simple structure and easy digital realization. However, with the signal amplitude a part of the loop control parameter in an SPD phasemeter, the controlling loop performance is unstable while the signal amplitude varies. The amplitude fluctuation cannot be effectively suppressed as a result. At the same time, due to the boundedness of the sinusoidal phase detector not exceeding −1 to +1, the phase detecting range is limited; therefore, the fast-acquiring range of the SPD phasemeter deteriorates.

To solve the above problems, here we report a phasemeter designed with a tangent characteristic phase detector (TPD). The output of the TPD is independent of the signal amplitude, which decouples from the loop control parameters and can suppress amplitude fluctuation effectively. In addition, the output range of the tangent signal is from −∞ to +∞. The theoretically infinite phase detection range allows the TPD phasemeter to have a larger fast-acquiring range than the SPD phasemeter. This paper is organized as follows. Firstly, the requirement for phase readout accuracy of 1 μrad/Hz^1/2^, preferably with a large lock range in GW detection, is introduced. Secondly, the principle of a traditional SPD phasemeter is introduced. Thirdly, the characteristics of the TPD phasemeter are calculated and show better performance than the SPD phasemeter, in theory. Fourthly, the noise equivalent phase (NEP) analysis shows that the TPD phasemeter has almost the same NEP parameter as the SPD phasemeter. Fifthly, to further verify the performances of the SPD and TPD phasemeters in FPGA, a digital fixed-point simulation is carried out. Finally, the conclusion and discussion are introduced. Both the TPD phasemeter and traditional SPD phasemeter show a good performance in phase tracking. The TPD phasemeter suppressed the amplitude fluctuation to the additive noise equivalent phase floor of 1 μrad/Hz^1/2^, which meets the requirements of GW detection. The TPD phasemeter has almost the same additive noise performance as the SPD phasemeter, verifying that the division operation introduces no more noise. Besides, the maximum lockable step frequency of the TPD phasemeter is almost three times that of the SPD phasemeter, which is a better tracking performance than the SPD phasemeter.

## 2. Principle of the SPD Phasemeter

**Figure 1:**
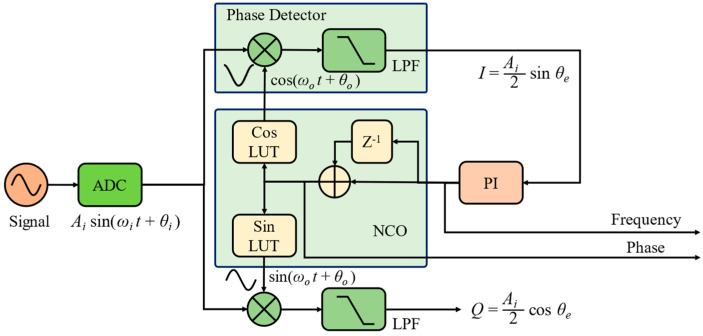
Principle of a phase-locked loop.

The principle of a PLL is depicted in Figure 1. The phasemeter consists of a phase detector (PD), a proportional integration (PI) controller, and a numerically controlled oscillator (NCO).

The NCO can output quadrature cosine and sinusoidal signals based on the input of the PI controller. The input signal is multiplied with the local sinusoidal and cosine waves and then low-pass filtered to obtain the *I*-branch and *Q*-branch signals, called *I*/*Q* demodulation,

(1) $$\begin{array}{l} I=A_{i}\sin(\omega_{i}t+\theta_{i})\cdot \cos(\omega_{o}t+\theta_{o})=\frac{A_{i}}{2}\sin\theta_{e}\\ Q=A_{i}\sin(\omega_{i}t+\theta_{i})\cdot \sin(\omega_{o}t+\theta_{o})=\frac{A_{i}}{2}\cos\theta_{e} \end{array}\;\text{(after LPF)}$$

where $\theta_{e}=\Delta \omega t+\theta_{i}-\theta_{o}$ and $\Delta \omega =\omega_{i}-\omega_{o}$. When the phase error is small, the *I*-branch’s output can be linearized to $u_{PD}=K_{d}\theta_{e}$ and is subsequently sent to the PI controller to tune the frequency of the NCO.

The output phase $\theta_{o}$ of the NCO is finally locked to the input phase $\theta_{i}$; then, $\theta_{o}$ is a measure of $\theta_{i}$. In an SPD phasemeter, $K_{d}=A_{i}/2$. The transfer function of the proportional integer (PI) controller is shown in Equation (2).

(2) $$H_{\mathrm{PI}}=\frac{\tau_{2}s+1}{\tau_{1}s}$$

where $\tau_{1}$ and $\tau_{2}$ are the controlling parameters. *s* is a symbol of the Laplace transform of continuous signals in the s-domain, while *z* represents the discrete signal transform. The commonly used transformation relationship between *s* and *z* is the Tustin transformation, shown in Equation (3).

(3) $$\begin{array}{l} s=\frac{2}{T}\frac{1-z^{-1}}{1+z^{-1}}\\ z=\frac{1+\frac{T}{2}s}{1-\frac{T}{2}s} \end{array}$$

where *T* is the sampling time of the discrete system. Thus, the open-loop and the closed-loop transfer functions of the PLL would be

(4) $$\begin{array}{ll} H_{open} & =K_{d}\frac{\tau_{2}s+1}{\tau_{1}s^{2}}\\ H_{close} & =\frac{K_{d}\frac{\tau_{2}}{\tau_{1}}s+\frac{K_{d}}{\tau_{1}}}{s^{2}+K_{d}\frac{\tau_{2}}{\tau_{1}}s+\frac{K_{d}}{\tau_{1}}}\\ & =\frac{2\xi \omega_{n}s+\omega_{n}^{2}}{s^{2}+2\xi \omega_{n}s+\omega_{n}^{2}} \end{array}$$

Equation (4) represents a basic second-order control system, where $\omega_{n}$ is the undamped natural frequency, and ξ is the damping factor, as shown in Equation (5).

(5) $$\begin{array}{c} \omega_{n}=\sqrt{\frac{K_{d}}{\tau_{1}}}\\ \xi =\frac{\tau_{2}}{2}\sqrt{\frac{K_{d}}{\tau_{1}}} \end{array}$$

**Figure 2:**
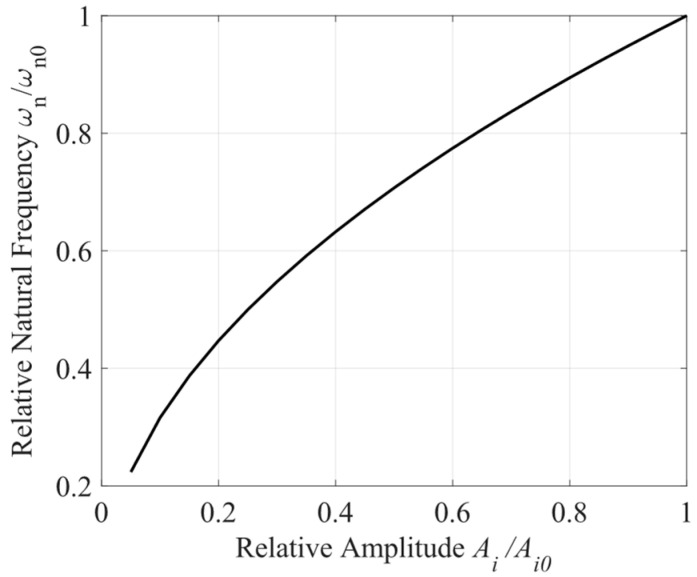
The relationship between relative natural frequency $\omega_{n}/\omega_{n0}$ and relative amplitude $A_{i}/A_{i0}$.

The signal amplitude, *A_i_*, forms a part of the loop-controlling parameter. The relationship between the relative natural frequency, $\omega_{n}/\omega_{n0}$, and the relative amplitude, *A_i_*/*A_i_*_0_, can be seen in Figure 2, where $\omega_{n0}$ and $A_{i0}$ are the initial values, respectively. Since the value of $\omega_{n}$ and ξ differ by a constant coefficient $\tau_{2}/2$, their relationships with the amplitude, *A_i_*, are basically the same. Both natural frequency and the damping factor decrease with the decay in the amplitude, which degrades the performance of the phasemeter, especially in the face of large variations in signal amplitude. Therefore, a phasemeter independent of the signal amplitude should be an optimum choice.

**Figure 3:**
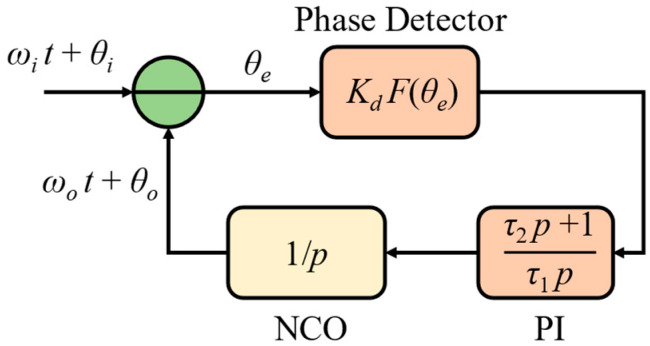
Phase domain diagram of phasemeter.

Furthermore, the nonlinear characteristics of the phase detector must also be taken into consideration for the locking performance, especially the boundary between lock and loss of lock. The dynamic function of the PI controller can be expressed as in Equation (6)

(6) $$H_{\mathrm{PI}}(p)=\frac{\tau_{2}}{\tau_{1}}+\frac{1}{\tau_{1}p}$$

where *p* is the differential operator characterizing $\frac{d}{dt}$. Thus, the phase domain diagram of the phasemeter can be shown in Figure 3, where $F(\theta_{e})$ is the function of the phase detector.

For ease of understanding, we define

(7) $$\begin{array}{l} \theta_{1}=(\omega_{i}-\omega_{o})t+\theta_{i}=\Delta \omega t+\theta_{i}\\ \theta_{2}=\theta_{o} \end{array}$$

The dynamic equation of the output phase and the residual phase error of the phasemeter can be simplified as

(8) $$\begin{array}{l} \theta_{2}=K_{d}\frac{H_{PI}(p)}{p}F(\theta_{e})\\ \theta_{e}=\theta_{1}-\theta_{2} \end{array}$$

The instantaneous phase error difference of the phasemeter can be written as

(9) $$\begin{array}{ll} p\theta_{e} & =p\theta_{1}-K_{d}H_{PI}(p)F(\theta_{e})\\ & =\Delta \omega +p\theta_{i}-K_{d}H_{PI}(p)F(\theta_{e}) \end{array}$$

where $p\theta_{i}$ is the phase differentiation of the input signal, which equals zero for a stable input. Thus, in the phase-locked state, $p\theta_{e}=0$, we see

(10) $$\Delta \omega =K_{d}H_{PI}(p)F(\theta_{e})$$

which indicates that the maximum allowable frequency difference is related to the signal amplitude in *K_d_*, the PI controller, and the characteristic of the phase detector. SPD phasemeter has $F(\theta_{e})=\sin\theta_{e}$. Thus, the signal after the PI controller in an SPD phasemeter would be

(11) $$\begin{array}{ll} u_{PI,SPD} & =\frac{\tau_{2}}{\tau_{1}}F(\theta_{e})+\frac{1}{\tau_{1}}\int F(\theta_{e})dt\\ & =\frac{\tau_{2}}{\tau_{1}}\sin\theta_{e}-\frac{1}{\tau_{1}\omega_{e}}\cos\theta_{e} \end{array}$$

where $\omega_{e}$ is much larger for locking, meaning the proportional item can be left alone, and Equation (10) can be simplified to

(12) $$|\Delta \omega |\approx K_{d}\frac{\tau_{2}}{\tau_{1}}|\sin\theta_{e}|\leq K_{d}\frac{\tau_{2}}{\tau_{1}},\;\mathrm{with}\;|\sin\theta_{e}|\leq 1$$

Equation (12) indicates a maximum allowable frequency difference between the input heterodyne tone and the local NCO, which means that $\theta_{e}$ within $\left[-\frac{cycle}{2},\frac{cycle}{2}\right]$ or cycle slips occurred. The frequency is thus called the fast-acquiring range, which is $|\Delta \omega_{SPD}|\approx K_{d}\frac{\tau_{2}}{\tau_{1}}$ for the SPD phasemeter. The fast-acquiring range is also proportional to the gain in phase detector *K_d_*. Therefore, the gain in the SPD is coupled into the loop control parameters, reducing the loop stability of the PLL. Thus, a PD with the gain independent of the amplitude of the heterodyne tone and a larger fast-acquiring range is more suitable for GW detection.

## 3. Characteristics of TPD Phasemeter

**Figure 4:**
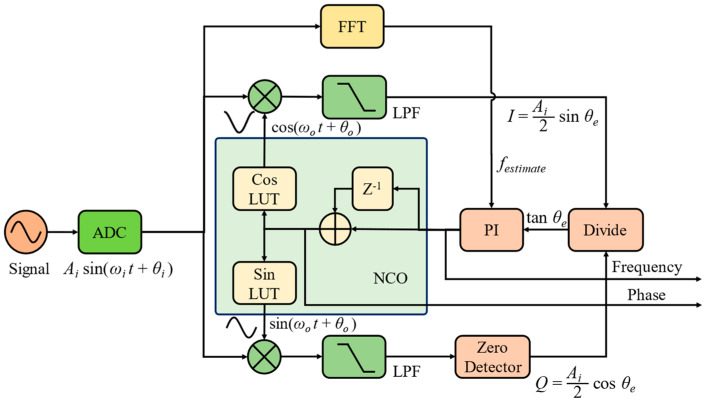
Schematic diagram of the phasemeter with tangent phase detector.

To meet the above requirements, a tangent phase detector is proposed in this manuscript, as shown in Figure 4. The TPD output increases rapidly when the phase error deviates from the balance point of zero phase error, which increases the feedback response speed. To maintain the stability of the system, especially when there are large disturbances, a fast Fourier transform is performed to estimate the frequency, *f_estimate_*, of the heterodyne tone. Due to the wide spectrum range of large disturbances, it will not affect the peak of the signal spectrum. A threshold is set near the peak frequency so that the PLL is not regulated beyond that threshold. When the Q-branch output is zero in division operation, the TPD will run to the pole of π/2. Therefore, a zero detector is settled in the program, and a value of 1 LSB will be set instead of zero for output. In our simulation, the 1 LSB corresponds to 6 × 10^−8^ with a 24-bit fraction width, which is too small to affect the simulation. Besides, the pole at π/2 is an imbalance point, which will not remain long and will be dragged back to the balance point.

The tangent phase error is derived from dividing *I* by *Q*.

(13) $$\frac{I}{Q}=\frac{\frac{A_{i}}{2}\sin\theta_{e}}{\frac{A_{i}}{2}\cos\theta_{e}}=\tan\theta_{e}$$

**Figure 5:**
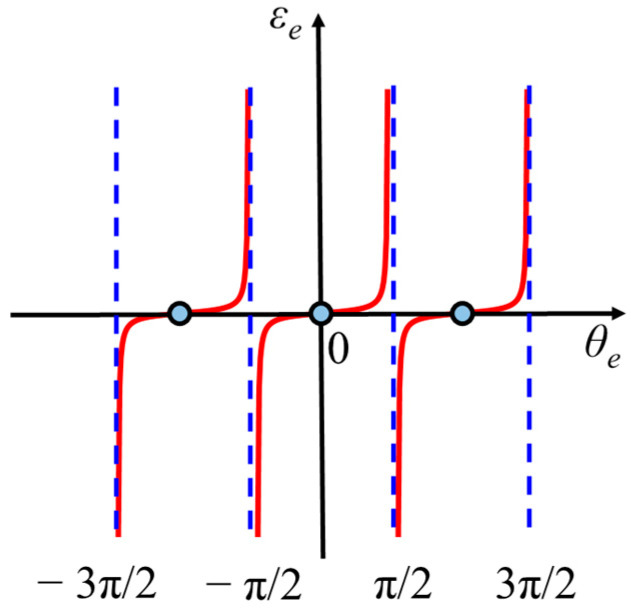
Characteristics of tangent phase detector.

The characteristics of the tangent phase detector (TPD) are depicted in Figure 5, where $\varepsilon_{e}$ is the error output of the TPD. Similarly, when the phasemeter is locked, $\theta_{e}$ is rather small, and there is $\tan\theta_{e}\approx \theta_{e}$, then the gain of the TPD would be $K_{d,TPD}=1$. The main difference from the SPD is that the gain in the TPD is independent of the amplitude of the heterodyne tone. This decouples the loop control performance of the TPD phasemeter from the input heterodyne tone’s amplitude. Thus, the TPD phasemeter will be insensitive to amplitude fluctuation, which is beneficial for reducing the noise of GW detection in the low-frequency band. Moreover, when the phase error deviates from the balance point, the output of the TPD will increase dramatically, making the feedback loop return to a controlled state.

Considering the fast-acquiring range for the TPD phasemeter, the output of the PI controller would be

(14) $$\begin{array}{ll} u_{PI,TPD} & =\frac{\tau_{2}}{\tau_{1}}F(\theta_{e})+\frac{1}{\tau_{1}}\int F(\theta_{e})dt\\ & =\frac{\tau_{2}}{\tau_{1}}\tan\theta_{e}-\frac{1}{\tau_{1}\omega_{e}}\ln|\cos\theta_{e}| \end{array}$$

It can be easily seen that the output of the PI controller would be (−∞,+∞) with residual phase error $\theta_{e}$ varying within $\left(-\frac{\pi }{2},+\frac{\pi }{2}\right)$, which allows the frequency difference to be infinity with $\theta_{e}$ variations in a cycle, and a stronger control to lock the input phase. Thus, the fast-acquiring range of the TPD phasemeter can reach infinity $|\Delta \omega_{TPD}|=\infty$ theoretically, which is much larger than the SPD phasemeter, indicating a potential for a greater fast-acquiring range.

## 4. Noise Equivalent Phase Analysis

For a phase readout system, the noise equivalent phase (NEP), which limits the noise floor of the phase readout system parameter, is important. Whether division operation in the TPD phasemeter will increase the noise for phase measurements or not must be analyzed. Taking the heterodyne tone with noise into consideration, as only noise near the carrier frequency affects the accuracy of the PLL, the input signal can be expressed as Equation (15).

(15) $$y=A_{i}\sin(\omega_{i}t+\theta_{i})+n_{c}\cos\omega_{i}t-n_{s}\sin\omega_{i}t$$

where *n_s_* and *n_c_* are the noise amplitude of the sinusoidal and cosine components, respectively. *n_s_* is unrelated to *n_c_*.

The outputs after *I/Q* demodulation can be expressed as

(16) $$\begin{array}{l} I=y\cdot \cos(\omega_{i}t+\theta_{o})=\frac{A_{i}}{2}(\sin\theta_{e}+\frac{n_{s}}{A_{i}}\sin\theta_{o}+\frac{n_{c}}{A_{i}}\cos\theta_{o})\\ Q=y\cdot \sin(\omega_{i}t+\theta_{o})=\frac{A_{i}}{2}(\cos\theta_{e}+\frac{n_{c}}{A_{i}}\sin\theta_{o}+\frac{n_{s}}{A_{i}}\cos\theta_{o}) \end{array}$$

To simplify, we can define two new noise items as Equation (17) shows.

(17) $$\begin{array}{l} n_{1}^{\prime }=\frac{n_{s}}{A_{i}}\sin\theta_{o}+\frac{n_{c}}{A_{i}}\cos\theta_{o}\\ n_{2}^{\prime }=\frac{n_{c}}{A_{i}}\sin\theta_{o}+\frac{n_{s}}{A_{i}}\cos\theta_{o} \end{array}$$

Therefore, the output of the *I*-branch and *Q*-branch can be simplified to the form of signal and noise superposition as

(18) $$\begin{array}{l} I=\frac{A_{i}}{2}(\sin\theta_{e}+n_{1}^{\prime })\\ Q=\frac{A_{i}}{2}(\cos\theta_{e}+n_{2}^{\prime }) \end{array}$$

The autocorrelation of the two new noise items can be expressed as

(19) $$\begin{array}{ll} E[n_{1}^{\prime }(t_{1})n_{1}^{\prime }(t_{2})] & =\frac{1}{A_{i}^{2}}\{E[n_{s}(t_{1})n_{s}(t_{2})]E[\sin^{2}\theta_{o}]+E[n_{c}(t_{1})n_{c}(t_{2})]E[\cos^{2}\theta_{o}]\\ & +(E[n_{c}(t_{1})n_{s}(t_{2})]+E[n_{s}(t_{1})n_{c}(t_{2})])E[\sin\theta_{o}\cos\theta_{o}]\}\\ E[n_{2}^{\prime }(t_{1})n_{2}^{\prime }(t_{2})] & =\frac{1}{A_{i}^{2}}\{E[n_{c}(t_{1})n_{c}(t_{2})]E[\sin^{2}\theta_{o}]+E[n_{s}(t_{1})n_{s}(t_{2})]E[\cos^{2}\theta_{o}]\\ & +(E[n_{c}(t_{1})n_{s}(t_{2})]+E[n_{s}(t_{1})n_{c}(t_{2})])E[\sin\theta_{o}\cos\theta_{o}]\} \end{array}$$

$R_{nc}=R_{ns}$, therefore,

(20) $$R_{n_{1}^{\prime }}(\tau )=R_{n_{2}^{\prime }}(\tau )=\frac{1}{2A_{i}^{2}}[R_{ns}(\tau )+R_{nc}(\tau )]=\frac{R_{nc}(\tau )}{A_{i}^{2}}$$

The autocorrelation of the two new noise items is reduced by $A_{i}^{2}$ times.

Obviously, $n_{1}^{\prime }$ is the NEP for the SPD phasemeter as Equation (21) shows.

(21) $$\begin{array}{ll} I & =\frac{A_{i}}{2}(\sin\theta_{e}+n_{1}^{\prime })\\ & \approx \frac{A_{i}}{2}(\theta_{e}+n_{1}^{\prime })\;(\sin\theta_{e}\approx \theta_{e},\;\theta_{e}\;\mathrm{is}\;\mathrm{quite}\;\mathrm{small}) \end{array}$$

Assuming the additive voltage noise spectrum to be $S_{V}=N_{0}\;(V^{2}/\mathrm{Hz})$, the NEP baseband spectrum can be obtained according to the correspondence between the autocorrelation function and the power spectral density (PSD)

(22) $$S_{n_{1}^{\prime }}=\frac{2N_{0}}{A_{i}^{2}}$$

Since the power of a sinusoidal signal is $P_{s}=\frac{A_{i}^{2}}{2}$, Equation (22) can be simplified to

(23) $$S_{n_{1}^{\prime }}=\frac{N_{0}}{P_{s}}$$

Equation (23) shows the PSD relationship between additive noise and phase, which is proportional to the noise power spectrum and inversely proportional to the power of the signal.

For the TPD output $u_{TPD}=\frac{I}{Q}$, the noise contribution can be expressed as:

(24) $$\begin{array}{ll} \frac{\delta u_{TPD}}{u_{TPD}} & =\frac{\delta I}{I}-\frac{\delta Q}{Q}\\ \delta u_{TPD} & =\tan\theta_{e}(\frac{n_{1}^{\prime }}{\sin\theta_{e}}-\frac{n_{2}^{\prime }}{\cos\theta_{e}})\\ & =\frac{1}{\cos\theta_{e}}(n_{1}^{\prime }-n_{2}^{\prime }\tan\theta_{e})\\ & \approx n_{1}^{\prime }\;(\theta_{e}\;\mathrm{is}\;\mathrm{quite}\;\mathrm{small}) \end{array}$$

When the loop is locked, or when the phase error, $\theta_{e}$, is quite small, the NEP of the TPD is the same as that of the SPD, and they will have the same phase noise response. This shows that the division operation of TPD will not raise the noise floor of the phase readout.

## 5. Digital Fixed-Point Simulation

In GW detection, the phasemeter is implemented in an FPGA, which processes data in a fixed-point mode with a limited processing rate. Therefore, the calculation accuracy and data representation range are also limited. Thus, the digital fixed-point simulation allows us to explore the performance between the SPD and TPD phasemeters. The same heterodyne tone is input to both SPD and TPD phasemeters, and the results are saved to be compared. The parameters of the digital fixed-point simulation are shown in Table 1. For the phase noise requirement of 1 μrad/Hz^1/2^, the bit widths of the ADC and LUT should be at least 8 bits. Thus, both the 16-bit width of the ADC and the 12-bit of the LUT meet the requirement of the GW detection.

The division of *I* and *Q* will be implemented in an FPGA with an IP core. It is dedicated to the calculation of fixed-point division operations, which can be achieved by setting the input bit width, output bit width, and fraction width. A division operation with the parameters listed in Table 1 is also deployed in an FPGA selected for GW detection, and the resource consumption results show that LUT resources consumed only 0.54%, while flip-flop resources consumed less than 0.01%. Therefore, division operation will not take up much FPGA resources. In the simulation, we set the truncation with reference to the IP core and verified the calculation results of the two methods, and the results show that their calculation results are consistent. To investigate the amplitude fluctuation suppression performance of the TPD phasemeter, a sinusoid modulation of the amplitude is added, with phase modulation to check the tracking performance of the two phasemeters. The input signal thus can be expressed by Equation (25),

(25) $$y_{in}=[V_{0}+V_{a}\sin(2\pi f_{a}t)]\sin[2\pi f_{0}t+\phi_{m}\sin(2\pi f_{m}t)]+W_{n}$$

where *V_0_* = 0.5 V, *V_a_* = 0.25 V, *f_a_* = 20 kHz, *ϕ_m_* = 0.1 rad, *f_m_* = 3 kHz, and *W_n_* is the Gaussian white noise equivalent phase noise of 1 μrad/Hz^1/2^.

**Figure 6:**
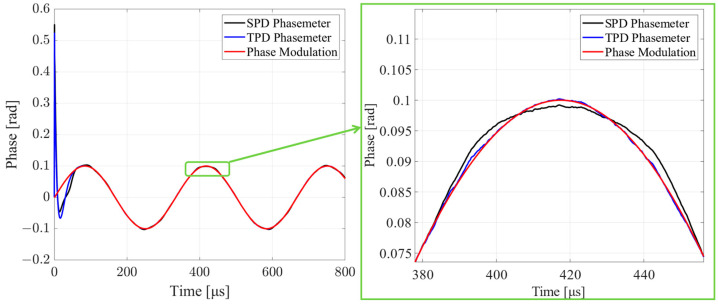
Comparison of the time domain results. The black and blue curves represent the measurement values of the SPD and TPD phasemeters. Both SPD and TPD phasemeters can track the sinusoidal phase modulation. The SPD phasemeter is distorted.

**Figure 7:**
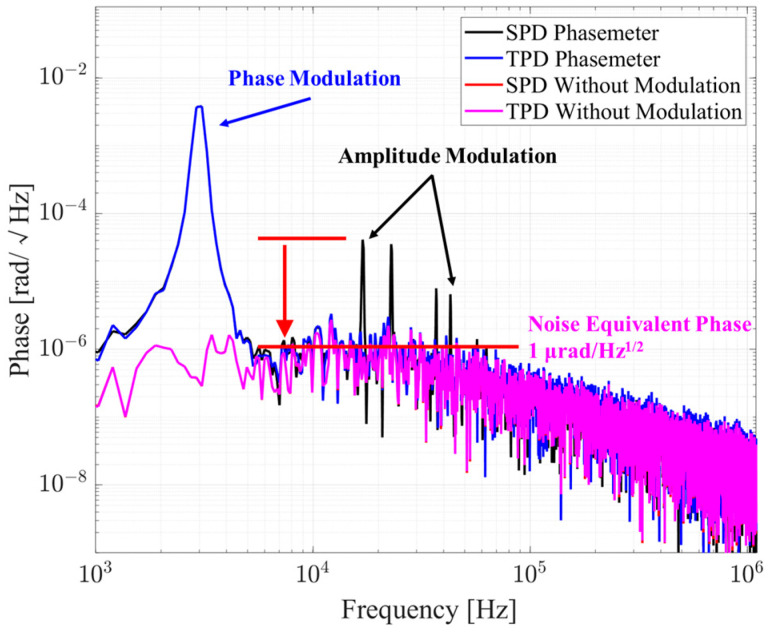
Comparison of the amplitude spectrum density. The black and blue lines stand for the results of the SPD and TPD phasemeters with phase modulation, amplitude modulation, and additive noise. The red and pink lines stand for the results of the SPD and TPD phasemeters with only additive noise, and they basically coincide. Peaks on the black curve between 10 kHz and 100 kHz come from the sum-frequency and difference-frequency of the phase and amplitude modulation frequencies. These peaks introduced by amplitude modulation are effectively suppressed to the noise floor by the TPD phasemeter.

The simulation results of the SPD and TPD phasemeters are shown in Figure 6 and Figure 7. In Figure 6, the time domain outputs of the two methods are compared. Both the SPD and TPD phasemeters can track sinusoidal phase-modulated signals with an amplitude of *ϕ_m_*. The TPD phasemeter can maintain phase measurement accuracy, while the SPD phasemeter output has a distortion. The chaos in the SPD and TPD phasemeters is due to additive Gaussian white noise, *W_n_*.

The amplitude spectrum density (ASD) in Figure 7 shows that both the SPD and TPD phasemeters perform almost the same with the phase modulation. Amplitude modulation coupled with phase modulation produces sum-frequency and difference-frequency signal peaks. The SPD phasemeter shows poor suppression of the amplitude modulation, while the TPD phasemeter suppresses the amplitude fluctuation to the noise floor of 1 μrad/Hz^1/2^, which fully meets the requirements of GW detection. In addition, the NEP of the SPD and TPD phasemeters basically coincide with the noise floor of 1 μrad/Hz^1/2^, which verifies the NEP analysis result that division operation will not induce larger noise.

**Figure 8:**
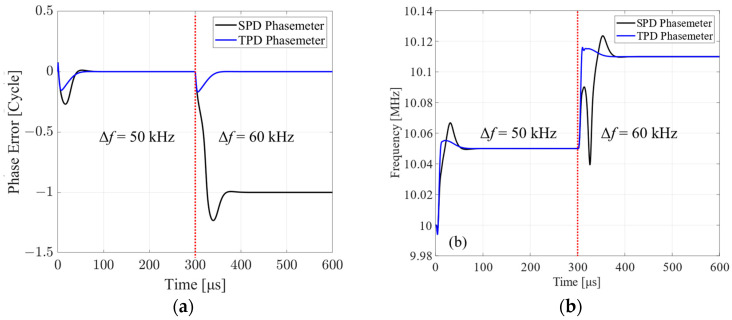
(**a**) Phase error of the SPD and TPD phasemeters with step frequency difference. (**b**) Frequency responses of the SPD and TPD phasemeters with step frequency difference. With a frequency step of 50 kHz, both the SPD and TPD phasemeters track the phase well. With a frequency step of 60 kHz, the SPD phasemeter induces a phase error of 1 cycle, called the cycle slip. The corresponding frequency response of the SPD phasemeter also shows a cycle of ups and downs when the step frequency is beyond the fast-acquiring range.

To test the fast-acquiring range of SPD and TPD phasemeters, a frequency step response simulation is implemented. The frequency difference between the input signal and NCO is set at 50 kHz at the beginning and 60 kHz at 0.3 ms. The phase tracking error and the frequency response of the two phasemeters are shown in Figure 8. It can be seen that the phase tracking error of the TPD phasemeter at the 50 kHz and 60 kHz frequency steps is controlled to be zero when stable. Meanwhile, the SPD phasemeter slips a cycle when tracking the 60 kHz frequency step. The frequency tracking performance shows a similar result, that the SPD phasemeter cannot respond quickly to the 60 kHz frequency step.

A further fine simulation shows that the maximum lockable step frequency of the SPD and TPD phasemeters are 55 kHz and 180 kHz, respectively. The reason for the finite fast-acquiring range is that the TPD phasemeter runs on a fixed-point processing system with a limited sampling rate. The fixed-point processing with limited bit width and calculation accuracy makes the result of the tangent operation also finite. The finite sampling rate also limits the feedback tuning speed, resulting in a phase error that cannot be controlled within ±π/2. The maximum lockable step frequency of the TPD phasemeter is still three times that of the SPD phasemeter, which shows a larger fast-acquiring range.

## 6. Conclusions and Discussion

A phasemeter as the readout system of the inter-satellite interferometer in a space-borne GW detector requires not only high accuracy but also insensitivity to amplitude fluctuation and a large fast-acquiring range. In the SPD phasemeter, the input signal amplitude participates in the controlling loop, worsening the loop stability and amplitude noise-suppression performance. The boundedness of the sinusoid function also limits the fast-acquiring range of the phasemeter. In contrast, the TPD phasemeter decouples from the amplitude of the heterodyne tone, making it perform well in amplitude noise suppression. Besides, the boundless output characteristic of the TPD brings to the phasemeter an infinite fast-acquiring range, in theory.

The digital fixed-point simulation shows that both the SPD and TPD phasemeters can track the phase modulation. However, the SPD phasemeter does not perform as well in suppressing amplitude fluctuation noise as the TPD phasemeter. The TPD phasemeter even suppresses amplitude fluctuations of 50% to the noise floor of 1 μrad/Hz^1/2^, which meets the requirements of GW detection. Furthermore, the maximum lockable step frequency of the TPD phasemeter is also three times larger than the SPD phasemeter.

Due to the finite bit width, calculation accuracy, and sample frequency, the resulting bit width of the tangent operation and feedback tuning speed are also finite, thus limiting the fast-acquiring range. The accurate fast-acquiring range of the TPD phasemeter is In addition, the low pass filter and division operations in the TPD phasemeter need more consideration. These issues are being further analyzed, and experimental verification is also being carried out.

## Figures and Tables

**Table 1:** Parameter of the digital fixed-point simulation system.

Parameter	Value
Sampling Rate	125 MSPS
Bit Width of ADC	16 bits
Bit Width of Sin/Cos LUT	12 bits
Heterodyne Frequency	10 MHz
Amplitude of Signal	0.5 V
Input Range of ADC	2 Vpp
Fraction Width of Tangent Operation	24 bits
Integer Width of Tangent Operation	24 bits
Fraction Width of Sinusoid Operation	24 bits
Integer Width of Sinusoid Operation	4 bits
Additive Gaussian White Noise (Equivalent Phase Noise)	1 μrad/Hz^1/2^

## Data Availability

Data are contained within the article.
